# Core competencies of peer workers who use pulse oximeters to supplement their overdose response in British Columbia

**DOI:** 10.1371/journal.pone.0273744

**Published:** 2022-09-02

**Authors:** Zahra Mamdani, Damian Feldman-Kiss, Sophie McKenzie, Mike Knott, Fred Cameron, Rayne Voyer, Jessica van Norren, Tracy Scott, Bernie Pauly, Jane A. Buxton

**Affiliations:** 1 BC Centre for Disease Control, Vancouver, BC, Canada; 2 Island Medical Program, Faculty of Medicine, University of British Columbia, Victoria, BC, Canada; 3 SOLID Outreach Society, Victoria, Canada; 4 RainCity Housing, Vancouver, BC, Canada; 5 Canadian Institute for Substance Use Research, University of Victoria, Victoria, BC, Canada; 6 School of Population and Public Health, University of British Columbia, Vancouver, BC, Canada; Kwantlen Polytechnic University Faculty of Science and Horticulture, CANADA

## Abstract

**Introduction:**

Peer workers (those with lived/living experience of substance use) are at the forefront of overdose response initiatives in British Columbia, Canada. The onset of the coronavirus disease pandemic has significantly compounded the impact of the overdose crisis. Peer workers are integral in supporting people who use substances. However, despite the important work they do, peer workers often lack formalized credibility and do not have the same resources available to them as service providers without lived experience. The peer-led project titled the Peer2Peer Project implemented several support programs for peer workers, including providing pulse oximeters to peer workers to supplement their overdose response procedures.

**Materials and methods:**

This study was a component of a larger evaluation of the pulse oximeter program at two organizations in BC. The study aims to highlight the competencies of peer workers who use pulse oximeters. Telephone interviews were conducted with seven peer workers who were given pulse oximeters. The transcripts were thematically coded using Covert et al.’s framework of core competencies of community health workers to compare our sample with other widely recognized professions.

**Findings:**

We found that peer workers who used pulse oximeters described several core competencies in their work and these were aligned with Covert et al.’s core competencies for community health workers, including assessment, community health practice, communication, diversity and inclusion, professional practice, and disease prevention and management.

**Conclusion:**

By aligning peer workers’ skills to those of community health workers, we create awareness on the competencies of peer workers in using oximeters to supplement overdose response and advocate for them to receive more recognition and respect within the workplace. Further, our findings act as groundwork for future research in identifying the professional proficiencies of peer workers.

## Introduction

In April 2016, the province of British Columbia declared a public health emergency due to the escalating rate of illicit drug toxicity deaths [1]. Peer workers, often referred to as “peers”, are at the forefront of overdose response initiatives in BC [2, 3]. Peer workers are individuals with lived/living experience of substance use who use that experience to inform their professional work [4]. Peer workers are employed in a variety of settings; these include shelter and housing agencies, stand-alone supervised consumption sites, and overdose prevention services, which are formal or informal sites equipped to respond to overdoses when they occur [5–8]. Peer workers perform a variety of services, including the distribution of harm reduction supplies, peer witnessing of substance use, providing referrals to resources, advocacy, outreach, overdose response, and research [9]. Peer workers often act as a critical link between available health and harm reduction strategies and the people they are intended to serve [10], thereby, increasing the accessibility and acceptability of programs for PWUS [4]. Employing peer workers in harm reduction programming has also been shown to improve health outcomes after an overdose event [11]. Furthermore, the robust interpersonal relationships between peers and the people they support enable the effective delivery of services [12–14].

Over time, the importance of peer workers in harm reduction efforts has amplified due to the rise in number of drug toxicity deaths as well as the increased poisoning of the illicit drug supply [7, 15]. The onset of the coronavirus disease of 2019 (COVID-19) pandemic has exacerbated the morbidity and mortality of the overdose epidemic. According to recent data from the BC Coroner’s Service, there were 2,224 suspected illicit drug toxicity deaths in 2021—which is the highest annual death toll on record [16]. Furthermore, there have been increasing reports in BC of substances containing mixtures of opioids and benzodiazepines and the identification of unregulated etizolam in urine drug screens [17–19]. Opioids mixed with benzodiazepines complicate management of acute opioid toxicity because while naloxone can reverse the opioid-mediated respiratory depression, it has no effect on the sedation caused by benzodiazepines [17–19]. Furthermore, this sedation often persists long after blood oxygen levels have returned to the normal range, which makes observer assessment challenging [17–19]. It is important to identify an overdose as it is happening and to respond quickly by giving rescue breaths to restore blood oxygen levels and administering naloxone to reverse the opioid-mediated effects [20]. The onus to respond to overdoses is generally placed on peer workers who are often at the scene of an overdose and well-positioned within their communities to respond [7, 13, 15].

Given the work performed by peer workers and their belongingness within the community that they support, a peer worker can be considered a community health worker defined as, “a frontline public health worker who is a trusted member of and/or has an unusually close understanding of the community served” [21]. Yet, unlike other community health workers, peer workers often lack recognition for their work and their unique skill set is often overlooked [15]. Previous research has highlighted that peer workers often have precarious work arrangements and are paid minimally, notably less than other front-line workers and support staff despite their similar duties [22–25]. Lack of peer worker recognition is a form of structural violence because it enforces the marginalization of PWUS and prevents them from achieving the same recognition as other workers. In this context, structural violence refers to the negative power of social institutions to perpetuate inequity to marginalized populations [26].

A pilot intervention called the ROSE model was created and implemented at some sites in BC to improve recognition, organizational supports, and skill development for peer workers [27]. This was done through the Peer2Peer project aimed at identifying, implementing and evaluating support interventions for peer workers in BC [28]. The project’s pilot sites were RainCity Housing in Vancouver, Coquitlam and Maple Ridge and SOLID Outreach in Victoria [27, 28]. One of the supports that were implemented was the provision of pulse oximeters to approximately 50 peer workers involved in overdose response at the pilot sites [27]. Pulse oximeters are small portable devices, usually applied to a finger, to non-invasively measure the oxygen saturation of blood [29]. This support aimed to aid decision-making regarding the need for providing rescue breaths and additional doses of naloxone when responding to an overdose [30, 31]. Not many peer workers have access to pulse oximeters; traditionally, pulse oximeters were considered a medical device used by clinicians and healthcare workers [32–34]. Equipping peer workers with pulse oximeters was an important step in recognizing them as health workers.

In this study, we aim to highlight the competencies of peer workers who use pulse oximeters and how their competencies align with those of community health workers [30]. The recognition of peer worker competencies is important in institutionally characterizing peer workers as experts and earning them the respect and recognition that they deserve. This paper will lay the groundwork for creating a standardized framework of core competencies for peer workers and make their roles and responsibilities visible.

## Materials and methods

### Objective

This study was a component of the larger evaluation of the pulse oximetry program at the Peer2Peer project’s pilot sites [35]. In this particular study, we sought to describe the competencies of peer workers who use pulse oximeters to supplement their overdose response in BC by mapping the data based on the competency domains set forth by Covert et al. (2019) for community health workers [21].

### Research design

The study incorporated a community-based research design, whereby peer workers are involved in all aspects of the study from the development of interview questions to data validation. The project team consists of academic researchers (ZM, DFK, SM, BP, JB) and peer research assistants (PRAs) from the pilot sites (MK, FC, RV, JVN, TS). The evaluation study received Research Ethics approval from the University of British Columbia Research Ethics Board (REB #: H18-00867-A005).

### Data collection and consent process

Data for the study was collected through qualitative telephone interviews with peer workers who were provided with pulse oximeters. Each site distributed the pulse oximeters to peer workers at different times when they appeared for their shifts. While all 50 peer workers at the pilot sites eventually had access to pulse oximeters, only 20 participants received their own based on a first-come-first-serve basis; the remainder of the pulse oximeters were kept in the head offices for use by all peer workers The recruitment was done by organizational managers and the PRAs at each site who reached out to peer workers who they knew had received their own pulse oximeters. Recruitment was done in person, via text message, or telephone on a rolling basis until data saturation was reached. Data saturation was determined when no new themes emerged from the data by conducting additional interviews. More details about this have been provided in the data analysis section.

If an individual expressed interest in participating, they received a consent form from the respective organizational manager. The consent form outlined, in accessible language, the study background, rationale, inclusion criteria, role of the participant, compensation, and potential risks and benefits. It also described what would happen with their data and how it would be used, how anonymity would be maintained, and who to contact for further questions or support. The consent form specified that participation was voluntary, and individuals had the right to refuse to participate or withdraw from the study at any time without needing to provide an explanation. Furthermore, the consent form indicated that declining to participate would not affect the services received from the respective organization, their employment, or their eligibility for future studies. The respective manager reviewed the consent form with the prospective participant, ensured comprehension, and elicited and answered any questions. The manager then scanned the signed and dated consent form to the project team, who contacted the participant to schedule the telephone interview. Participants were given a copy of their consent form for their records.

Interviews started with a brief description of the project and the goals of the interview, followed by an opportunity for the participant to ask questions. Conversations during the interview were directed by a semi-structured interview guide (supporting file), which was informed by the evaluation objectives and preliminary discussions with PRAs during the bi-weekly project team meetings. The interview guide was reviewed by three members of the academic research team and a PRA (DFK, ZM, JB, FC). The interview guide was adjusted after the first and second interview to improve the flow of the interview and remove redundant questions. The interviews were conducted by a second-year medical student (DFK, a co-author of this paper) as part of their second-year flexible enhanced learning course of the MD undergraduate program at the University of British Columbia (UBC). Among other goals, this course aims to foster scholarship, innovation, and community engagement among UBC medical students.

Each interview lasted approximately 45 minutes. The interviews were audio-recorded and each participant received $25 CAD in cash, as per the BC peer worker payment standards [36]. The audio recordings were transcribed verbatim by an external transcriptionist. To maintain participants’ anonymity, names were not elicited during the interviews, transcripts were de-identified before data analysis, and files were password protected.

### Data analysis

An initial read of the first three interview transcripts was conducted by two authors (DFK and ZM) to determine the key concepts discussed. As each additional interview was conducted, the transcript was reviewed by DFK to determine if any new concepts were emerging and whether additional interviews were required. After six interviews, no additional concepts were emerging; a seventh interview was conducted to confirm that data saturation had indeed been achieved.

Although the aim of the larger evaluation was to assess the uptake, utility, and impact of the pulse oximetry program, several questions in the interview guide required the participants to narrate the process and experience of using pulse oximeters during overdose response. During this initial review of first three interview transcripts, peer worker competencies emerged as a major concept from the evaluation. Therefore, for the reminder of the interviews, follow up questions were asked with this applied focus in mind. This modification of the interview guide according to emerging concepts is in line with the iterative processes of qualitative research [37]. During data analysis, particular attention was paid to the peer workers’ descriptions of their work to identify their skills and competencies. We decided to map the data to the competency domains set forth by Covert et al. (2019) for community health workers [21]. Criteria for selecting a framework included: 1) Similarity in the work with the professionals for whom the framework is intended, in this case, community health workers and 2) Similarity in work contexts. Given that peer workers who use pulse oximeters for overdose response work in public health settings, the descriptions of their work aligned with the roles of community health workers, specifically CHW1s, who, as described by Covert et al. (2019), work in community-based organizations. [21]. In other words, peer workers, like CHWs, are involved in providing health information, conducting outreach, promoting awareness, and facilitating access to services [4, 9, 10, 21].

Thematic analysis was used to identify, organize, and report themes [38, 39]. A coding framework was created based on Covert et al. (2019)’s domains; assessment, community health practice, communication, diversity and inclusion, professional practice, and disease prevention and management. The framework was inputted into NVivo (QSR International, version 12) where coding progressed in an iterative and reflexive manner. Our analysis was primarily deductive [40] in that the competencies described by the participants were sorted based on Covert’s domains.

The raw data was initially coded by two academic members of the research team (DFK and ZM). To minimize the loss of some important aspects of the reality of peer workers’ lives, participatory coding was employed in subsequent rounds and data validation meetings were conducted with PRAs. Particularly, after the initial coding and sorting of quotes into the relevant ‘buckets’ based on Covert et al. (2019)’s domains, representative quotes and the coding framework were brought to the PRAs for data validation during a team meeting. Through a discussion and consensus process, PRAs confirmed whether each quote had been classified in the correct bucket, as per the real-world context of the peer workers. The PRAs also selected quotes that are most reflective of their experience for presentation within this paper through a voting process.

## Results

A total of seven telephone interviews were conducted: five participants were from SOLID Outreach and two participants were from RainCity Vancouver. We have not provided the demographic characteristics of the participants of this study to avoid identifying them, however, we acknowledge that the sample from which the participants were selected consists of a range of genders, ages, and ethnicities; this information is presented in a separate evaluation report of the Peer2Peer project [41].

Table 1 indicates the competency domains as well as the associated competencies set forth by Covert et al. (2019) [21] that were described by our participants. We describe each competency in the section below.

**Table 1:** Competency domains and competencies from Covert et al. (2019) framework showcased by participants.

Competency Domain	Competencies described by participants
Assessment	• Identifying available sources of data • Using data to decide on interventions • Demonstrating program or intervention effectiveness with data
Community Health Practice	• Conducting community outreach • Facilitating clients’ access to health services and resources • Organizing client education opportunities • Advocating for clients’ needs
Communication	• Communicating with linguistic and cultural proficiency (e.g., in writing, orally, and visually) • Distributing health information to community members
Diversity and Inclusion	• Functioning without judgement, bias, and stereotype
Professional Practice	• Functioning as part of a health care team • Applying continuing education to work responsibilities • Incorporating ethical standards of practice into all interactions with individuals, organizations, and communities
Disease Prevention and Management	• Sharing information about prevention of overdoses and other negative health outcomes • Supporting continuous availability of health services to clients

### Assessment

As defined by Covert et al. (2019), the assessment domain is the application of “data to community health actions at client and community levels” [21]. We have conceptualized the term “data” as any piece of information collected during assessment that aids in determining an individual’s condition and the necessary course of action. This domain includes several competencies, including identifying available sources of data, using data to decide on interventions, demonstrating program or intervention effectiveness with data, and preparing reports [21]. Peer workers in our sample exhibited several of these competencies.

#### Identifying available sources of data

Many participants explained how they identified the visible cues of an overdose on the individual they were responding to, and how they used that data to make an assessment of the client’s condition. One participant described:

*“The first thing you do is observation on the individual that’s down*. *Look at his skin tone*, *skin colour*, *lip colour*. *If they’re leaned over on the table*, *look after any signs of whether the person’s breathing*. *And if they’re shallow breathers that’s when I usually take the [pulse] oximeter*, *put it on their finger and I can see what their oxygen level is*, *how their heart rate’s doing*. *I have to look at a lot of different things*.*”–*Participant 7, Victoria

The quote indicates the participant’s competency in making assessments by remarking on a client’s appearance, level of consciousness and responsiveness, quality of breathing, and vital signs such as oxygen saturation and heart rate.

#### Using data to decide on interventions

Some participants also explained how they determined the interventions to use based on the oxygen saturation of the person’s blood. A participant explained how they used a pulse oximeter to help assess a client’s condition to inform their interventions:

*“If someone’s on a stimulant and they’re going through a psychotic episode or something and they’re sitting in a chair sweating profusely and stuff like that*, *I just slap [the pulse oximeter] on*. *I want to see what’s going on with his heart*. *I want to see what’s going on with his breathing*. *And if he’s on down [opioids] I do the same thing*. *If I look at him and I can’t get a response off him*, *I’ll sit down*, *slap that one on and see his oxygen level*. *Find out whether I need to do breaths*, *whether I need to do Narcan*.*”–*Participant 7, Victoria

This quote indicated that participants used pulse oximeters to differentiate between overdoses caused by stimulants (which increase heart rate) [42] and opioids (which decrease heart rate) [43] and used this information to decide on the appropriate responses to each type of overdose.

The use of pulse oximeters to decide on interventions was further described by another participant:

*“I’d always put in […] a pulsimeter just so […] we had reasons to do what we did*. *Say it was under 90*, *or it was 65 oxygen level or whatever… and for me that [shows] you need oxygen*.*”—*Participant 1, Victoria

Similarly, participants discussed how the pulse oximeter allowed them to make early assessments about the person’s oxygen saturation which helped them intervene early, keeping in mind some of the nuances surrounding the choice of interventions (e.g., naloxone administration, calling 911, etc.):

*“Sometimes [clients will] come out of their [nod] and they get violent once you Narcan them*. *So*, *you don’t want that*. *If you can catch an overdose before it happens*, *in the beginning stages*, *when their brain is stopping communicating with their lungs*, *you just can remind them to breathe*. *[…] And just really stimulate them*, *and just get them breathing on their own*. *That can help so much*, *and that’s because of checking the [pulse oximeter]*. *Without the [pulse oximeter] I wouldn’t know that they needed that kind of oxygen*.*”–*Participant 4, Victoria

This participant discussed the importance of using data to intervene early in order to prevent the overdose from happening in the first place.

#### Demonstrating intervention effectiveness with data

Some participants described how the assessment conducted by peer workers, combined with the use of pulse oximeters, could prevent the unnecessary use of naloxone (often referred to as NARCAN®). In the words of one participant:

“*Before we had [the pulse oximeters] there [were] people getting Narcanned that didn’t necessarily need to be Narcanned*. *They were not overdosing*. *They were just sleeping*.*”–*Participant 5, Vancouver

#### Summary of the assessment domain

Overall, participants’ descriptions indicated that they have the competencies associated with the Assessment domain, especially in relation to identifying whether someone is experiencing an overdose and deciding on appropriate interventions. Through their assessments and interventions, participants seemed to improve the experience and outcomes for their clients and conserve valuable resources (such as naloxone if administered unnecessarily).

### Community health practice

The community health practice domain is defined as “implement[ing] health promotion strategies within communities” [21]. The associated competencies include facilitating clients’ access to health services and resources, conducting community outreach, organizing client education opportunities, and advocating for clients’ needs [21]. Participants in this study described many of these competencies.

#### Conducting community outreach and facilitating client access to health services

Our participants described being involved in a variety of roles, including conducting outreach and distributing harm reduction supplies in the community. One participant described:

*“I’ve worked the street handing out supplies to all the different parks*. *I’ve worked from [location 1] all the way out to [location 2]*, *all the different streets*. *I carry supplies for everything*: *feminine products to safety kits to joints*. *Sometimes*, *someone’s in a horrible mood or they’re not feeling quite right*. *[I hand out] DVD’s*. *I like the streets*, *that’s where the people are*.*”–*Interview 7, Victoria

This quote suggested the commitment of participants to meet people where they are at, thus improving access to services for PWUS.

#### Organizing client education opportunities

Participants also described providing health education to members of the community. For example, some participants described educating others on how to assess a client’s condition. One participant discussed how they used a pulse oximeter to provide client education:

*“And it’s just really handy for everybody not just myself*. *Say [someone’s] boyfriend might be overdosing*, *it’s handy for them*, *too*, *to see the numbers and explain to them*, *‘okay*, *this is what’s happening*. *Under 90 is bad*. *Over 90 is good’*. *And you can put it on their finger and show them*, *‘see you’re at 98*. *And so you’re alive’*.*”–*Participant 1, Victoria

This quote suggested that participants not only used their knowledge to respond to the situation at hand, i.e., reversing an overdose, but also took the extra step of providing education to those who support individuals who use substances.

Some participants mentioned that they provided naloxone training. In the words of one participant: “*I’ve taken all the Naloxone training and I’m a Naloxone trainer myself*.”–Participant 4, Vancouver

#### Advocating for clients’ needs

In addition to conducting outreach and providing health education, participants described serving as the voice of the community and advocating for health promotion programs for their clients. Participants shared that they were strongly embedded within the community of PWUS and had good understanding of the needs of the community. As one participant stated:

*“There are [people] that could benefit from a definite program [training]*. *Like a day [training] would be great*. *And if [the pulse oximeter program] was operating in the community*, *it would be a great thing for everybody to be able to just go and take one*.*”–*Participant 3, Victoria

Another peer worker described the need for a peer debriefing program for people with lived/ living experience:

*“[We need] another peer worker that’s designated to just hear us out*. *Some decompression after we’ve had to use [oximeters] and had to apply our experience [i*.*e*. *respond to an overdose]*. *Because with every experience that we have*, *we take a little something of that home with us*.*”–*Participant 6, Victoria

These examples show the commitment of participants to advocate for better training and resources for their colleagues as well as their clients.

#### Summary of the community health practice domain

Overall, within the domain of community health practice, what particularly stood out about peer workers was their genuine care and concern participants had for others. In the words of one participant:

*“And this [overdoses] is something that [I can’t take lightly]*. *These are my friends*, *my peers*. *I need to help them not get brain damage*. *So*, *it’s like*, *what’s my best course of action*? *How do I help them*?*”–*Participant 1, Victoria

As the above quote indicated, the close relationships that participants had with the members of their community encouraged them to take the extra step to protect their community members and keep them safe from harm.

The participants’ descriptions indicated that as members of the community of PWUS, participants seem to have a strong understanding of the needs of their community and are best suited to implement health promotion programs within their communities. Furthermore, participants seemed to have a strong relationship with their clients, which can facilitate trust and understanding.

### Communication

The communication domain is defined as “gather[ing] and exchang[ing] information with clients and community stakeholders.” The associated competencies include communicating with linguistic and cultural proficiency (e.g., in writing, orally, and visually), distributing health information to community members, and disseminating information about health programs [21]. Participants in this study described several of these competencies.

#### Communicating with linguistic and cultural proficiency

Participants described how they interacted with a variety of community members and professionals to manage life-threatening events. They mentioned the importance of not only having the knowledge and ability to respond to overdoses, as described in the earlier sections, but also the ability to communicate effectively with their clients, community members, and emergency medical services. One participant described how having a pulse oximeter to objectify data allowed them to talk confidently with their co-workers and other professionals:

*“Being able to quantify the effect of an overdose—it just changes everything*. *It changes the way that we speak and interact with each other*. *Not only that*, *but it gives us something to [discuss] with co-workers*. *You’ve now got information to give them and they know what that information means so they know how they can help*. *It changes the entire situation*. *It changes the dynamics between staff*. *It changes the dynamics between the person who’s overdosed and staff*.*”–*Participant 6, Victoria

Another participant described how using the pulse oximeters eased their communication with first responders:

“[*When] we’re using the oximeter now*, *is we have background knowledge when the paramedics get there as to what the number says*, *you know*, *what kind of trends there have been in the 15 minutes since we made the call*. *And they can react accordingly*. *So it’s very helpful in that sense*.*”–Participant 7*, *Victoria*

The quotes above highlighted the importance of having a tool to quantify participants’ assessments as it allowed them to better communicate and collaborate with their colleagues and other first responders.

Several participants mentioned how their communication style elicited positive reactions from their clients:

*“For the most part I’m extremely docile*. *[If] somebody wants to get mad at me*, *go ahead*. *I’ll probably calm them down by the time I’m done talking to them*. *[While responding to an overdose]*, *I just calmly coax them into breathing again*. *If you’re calm with them*, *they’re going to wake up calm*.*”–*Participant 5, Vancouver

This narrative indicates the sense of calmness of the participant. The participant suggested that when peer workers maintain a calm and composed nature, it can foster relationship-building and trust as well as have a calming effect on some of their clients, leading to positive reactions from their clients.

Shared experience allowed participants to interact with their clients in a manner that facilitated trust and respect:

*[Overdoses] almost never happen when [X peer worker] is [working] and he says that’s due to the way that he interacts with people*. *He uses responsibly himself*. *And he’s a respected member of the community*. *And people know better*. *And that’s very important because the level of accountability isn’t quite there in the way that it’s managed by a health authority or outside body*. *But having a member of the community there where [clients] are able to talk as equal*, *people take more responsibility for their actions and that shows in the number of [overdoses]*.–Participant 6, Victoria

This quote suggested that PWUS often trust peer workers more than other first-responders because of their shared lived/living experience which enables them to “*talk as equal(s)*”. Further discussion with the participant during data validation revealed that the participant tends to use non-stigmatizing and person-first language to elicit trust and respect from his clients. This points to the importance of using appropriate terminology while communicating with PWUS.

#### Distributing health information

Many participants described how they were involved in educating their community members on a variety of topics, which were described earlier under the section of “organizing client education opportunities”. In relation to communication, participants shared that they incorporated demonstrations and used language and metaphors that would be relatable to their clients, based on their own lived/living experience of substance use. For example, one participant described how they would explain to their client the need to drink more water, especially after using methamphetamine:

*“I’ll explain things like ‘listen*, *[from] when I was using*, *I understand dehydration and you’re sweating like a dog right now*. *You are losing water*. *You need to have some water’*.*”*—Participant 7, Victoria

#### Summary of the communication domain

Overall, participants described how their success in responding to overdoses was due to their ability to communicate effectively with various individuals, including the individuals they responded to as well as other professionals such as paramedics that they collaborate with to improve the outcomes for PWUS.

### Diversity and inclusion

The diversity and inclusion domain is defined as “respect[ing] the range of differences among individuals and communities” [21]. The associated competencies include functioning without judgement, bias, and stereotype, identifying health disparities within communities, and taking active steps to achieve health equity [21]. Participants in this study described several of these competencies.

#### Functioning without judgement, bias, and stereotype

Participants in this study shared that they often had the unique opportunity to serve clients with whom they shared lived/living experience. Participants mentioned that this shared experience allowed them to serve compassionately and empathetically which, in turn, in their opinion, led to working without judgement, bias, or stereotype. As described by a few participants:

*“That’s the nice thing about being a peer*. *We’ve all been in these people’s shoes before*. *So*, *I want to treat these people how I would want to be treated*. *And so*, *I’m going to try my best to do everything*.*”–*Participant 1, Victoria

*“When you show real concern for people*, *they feed off that*. *They see that and that’s how we work with people*. *I don’t want to be a stranger*. *I don’t want to be some guy up there*, *you know*, *just running and going by the facts*. *I talk to them*. *‘How you doing*? *How you feeling*? *You okay*?*’”—*Participant 7, Victoria

These quotes highlighted how shared lived/ living experience encouraged participants to provide judgement-free and empathetic supports to PWUS. Participants in this study described treating community members with respect, regardless of their background and history of substance use, and “as equals” as described earlier in the words of one participant.

### Professional practice

The professional practice domain is defined as “performing within an organization”. The associated competencies include adapting to multiple responsibilities, functioning as part of a health care team, applying continuing education to work responsibilities, and incorporating ethical standards of practice into all interactions with individuals, organizations, and communities [21].

#### Functioning as part of a health care team

Participants in this study consistently described the ability to function as part of a health care team, be it with their colleagues, the health authorities under which their organizations fall, or other professionals they encounter, such as paramedics. As one participant mentioned, *“Most of us had worked hand in hand with BC Ambulance”–*Participant 6, Victoria. Another stated:

*We are all here to help*. *[…] There [are] different roles to play*, *and we all play good roles in those roles*. *So*, *it’s just making sure people know how to best support people*, *I guess*, *and that’s just figuring out crowd control*, *911*, *who’s going to do the rescue breaths*, *who’s going to draw up the Narcan*.*”–*Participant 1, Victoria

As part of working in a team, participants recognized their role and the roles of other members of a health care team by acknowledging what they could bring to an overdose response, such as their lived/ living experience, which as described earlier, facilitated trust and understanding with their clients. Participants also described knowing their limitations and knowing when they needed to make referrals. In the words of one participant:

*“I think the main thing is—I know my limitations*. *That’s what’s really important too*. *What I can do and what I can’t do*. *That’s why paramedics are coming*.*”–*Participant 7, Victoria

During data validation, several participants described that while they were competent in responding to overdoses, they often needed to hand off to paramedics and other health professionals for more advanced measures such as supplemented oxygen through oxygen tanks, and further testing in hospitals.

#### Applying continuous education to work responsibilities

Another competency that participants described was the ability to apply continuing education to work responsibilities. This continuing education included naloxone training, first aid, pulse oximeter training, and being familiar with the data from local and regional drug checking services. For example, one participant discussed how they applied knowledge of current drug contaminants to their response to opioid overdoses:

*We have drug testers on site at one of the places*. *So*, *they always give updates about what kind of things are going around in the drugs*. *So*, *we can be mindful of that*. *Like say there’s an increase of benzos*, *so what do we know about benzos*, *and how do we move forward with the benzos in fentanyl overdose*?–Participant 1, Victoria

This quote suggested that participants, like other health care workers, kept themselves up to date in their knowledge and current drug alerts to share timely and appropriate information with their clients. They also seemed to have knowledge about other external services, such as availability of mental health services and support circles, so they could make appropriate referrals. Many participants described keeping abreast of current trends and required protocol for overdose response, especially in light of COVID-19 when peer workers had to familiarize themselves with the use of appropriate personal protective equipment and guidance regarding rescue breaths.

#### Incorporating ethical standards of practice

Additionally, participants showed competency in incorporating ethical standards of practice into all aspects of their work. An important example was their approach to naloxone administration. Participants described how naloxone can be lifesaving as an opioid antidote that temporarily reverses opioid-mediated effects, but how it can also cause withdrawal. They described that the decision to administer naloxone must be made with careful and ethical consideration. In the words of one participant:

*“We don’t want to sit down and get a guy up with Narcan when he goes into instant withdrawal*. *‘Cause then you got a whole new situation to deal with*.*”–*Participant 7, Victoria

The above quotes suggested that the unnecessary administration of naloxone can be considered unethical because it may result in the abrupt onset of significant pain, thus doing more harm than good. As such, the decision to administer naloxone should be made after careful consideration and assessment.

#### Summary of the professional practice domain

Many peer workers described that due to the knowledge, experience, and resources (such as pulse oximeters) they have, they are trusted by their clients. This, in turn, seemed to create a sense of professionalism and confidence among participants. One participant described:

*“[The pulse oximeter] almost has a calming effect and the person is okay*. *[…] I don’t know what it is*, *but it changes the way that we interact with the person*. *It gives*, *maybe*, *it’s a more professional feel*. *[…] Whereas*, *oftentimes*, *if you say a paramedic is on the way*, *for example*, *the person will just get up and leave the site immediately*.*”–*Participant 6, Victoria

As described in the latter part of this quote, PWUS seem to trust peer workers more than other professionals such as paramedics. This is perhaps due to the lived/living experience that peer workers share with their clients, which creates some sort of safety net. Overall, participants’ descriptions indicate that they showcase all the relevant competencies within the professional practice domain for CHW.

### Disease prevention and management

Covert et al. (2019) defines this domain as “implementing strategies to reduce the burden of preventable disease” [21]. However, this definition can be adapted to include all negative health outcomes, not just disease. In the context of overdose response, it would involve preventing overdoses. This domain would then include such competencies as facilitating referrals to prevent overdoses and other negative health outcomes, supporting continuous availability of health services to clients, and sharing information about prevention of overdoses and other negative health outcomes.

#### Sharing information about prevention of overdoses

Some participants described how they often prevented overdoses by educating clients to reduce the risk of overdose as well as by monitoring and intervening early:

“*I try to even monitor what the people are doing or where their head space is at and try and prevent the overdose in the first place*. *Rather than responding to it*.*”–*Participant 2, Victoria

This quote described the importance of monitoring closely and intervening early to prevent negative outcomes. Participants also mentioned that unlike emergency service providers, they often have insights into the types of substances that a person may have used due to their personal relationship with their clients. This allowed them to intervene fast and early when an overdose was likely to happen.

#### Supporting continuous availability of health services

In addition to preventing the onset of negative health outcomes, participants described supporting the continuous availability of health services to clients. As members of the community, participants often encountered situations and responded to overdoses even outside their regular work hours. As such, one participant suggested having a portable pulse oximeter with them at all times:

*“I would suggest that everyone should have their own [pulse oximeter]*. *It’s such a small expense when you consider what you get out of it*. *It’s roughly the cost of a tee-shirt*. *And then not only that but people are going home*, *and they probably see just as many overdoses when they’re not working as they do at work*. *So*, *it’s of great value*.–Participant 6, Victoria

Participants showed substantial altruism towards members of their community and appeared to put others first, but at the same time, they acted responsibly to prevent transmission of secondary infections. For example, with the onset of COVID-19, peer workers ensured they donned their personal protective equipment before responding to overdoses. One participant described:

*“Our interest is making sure that we can save the individual*. *And if it means me going inside*, *say*, *a tent to do something*, *first thing I’m doing*, *you know*, *I put a mask on*. *And put an N95 and I’ll make sure that that part is done*. *It’s a personal choice to me*. *I love what I do*. *And to me the priority is saving lives*.*”–*Participant 7, Victoria

Participants described how they are making a significant contribution to preventing overdoses in the province through their commitment and passion for saving lives:

*“I think I hit a hundred overdoses about three months after I got my formal training*. *And now I’m at 834*. *It’s one of those things that*, *I don’t know*, *I used to get excited when I saved somebody*, *and now it’s*, *like*, *a regular thing*.*”–*Participant 5, Vancouver

This quote illustrated how participants were playing a crucial role in changing the course of the overdose crisis in BC; by saving lives and preventing overdose deaths, participants are significantly reducing the overall burden of the overdose crisis.

## Discussion

Our research found that peer workers in our study described the six core competencies for community health workers established by Covert et al. (2019). The participants attributed their competencies to a combination of their lived/ living experience of substance use, the training they received from their respective organizations as well as they relationships and ability to build trust with the community they serve. Our findings were in line with several previous studies that have shown the effectiveness of peer-led assessment of overdoses and how that has led to improved health and social outcomes for those likely to experience an overdose [7, 9, 13, 44–46].

Peer workers in our study described how, like community health workers, they were integral to the delivery of community health services and health education. Past studies have indicated that peer workers are well-situated within the community of people likely to experience an overdose [8]. It was interesting to note an important distinction between peer workers and other community health workers and/ or emergency service providers in that peer workers don’t have to “identify stakeholders to support community outreach” and “engage community partners” as stated in Covert et al. (2019)’s paper [21]; peer workers are themselves the stakeholders and partners.

We found that participants’ connection with the community they support removed barriers to communication inherent in the interactions between health professionals and PWUS [4]. The effectiveness of peer workers’ communication skills seemed to be rooted in the deep sense of empathy for members of their community [10]. Peer workers tend to collaborate well with other service providers and communicate effectively using objective data from pulse oximeters to improve their community members’ chances of survival.

Similar to peer workers’ ability to effectively deliver community health practice due to their belonging within the community, we found that our participants described providing non-judgemental services to all and treated them “as equals”. This was consistent with other studies that indicated that peer workers are well-equipped to practice principles of diversity and inclusion [21]. This was not surprising given how peer workers tend to be familiar with the health disparities present within their communities, and are well aware of the tangible ways in which their health outcomes are shaped by broader systems that disadvantage them [8]. Like other studies that have indicated that peer workers interact with those they support in a trauma-and-violence-informed manner [8], we found that participants used appropriate non-stigmatizing and person-first language. In other words, as described in other studies, peer workers have often experienced judgement, bias, and stereotypes themselves, which makes them more likely to behave in a manner that mitigates these potential harms [24].

Peer workers in our study also described competency in professional practice. This was consistent with other studies which show how competent peer workers are in professional roles and how they are increasingly incorporated into various roles, such as but not limited to, research assistants, advisory committee members, and media production assistants [9]. Participants described their multi-sectorial involvement and their ability to operate and collaborate effectively as part of a team. They also showed their competence in adhering to ethical standards of practice especially in relation to the administration of naloxone and knowing how much would be too much. This was consistent with other studies which indicated that bystander-administered naloxone is safe and effective for opioid overdose reversal [47].

Lastly, our participants consistently described skills in disease prevention and management. This finding was in line with several studies that have shown that through education on safer substance use and distribution of safe supplies, peer workers have reduced the levels of infectious diseases such as HIV and hepatitis C within communities using substances [45, 46]. Furthermore, through systems navigation advice, they also facilitate access to critical health services offering treatment and prevention of health-related harms [9, 48, 49]. Most tangibly, peer workers have been integral in preventing overdoses and overdose deaths through the use of naloxone and overdose response techniques [11, 50].

Given peer workers’ contribution towards the opioid crisis in BC, in saving lives and preventing harm, peer workers deserve increased recognition for their expertise as well as ongoing training, support, and motivation to continue working in this otherwise stressful area of work. Developing a set of core competencies for peer workers is a step towards improved respect and recognition and may also serve as a motivator to continue working in overdose prevention settings. Organizations must also strive to empower peer workers in leadership roles and continue to engage peer workers in decision-making and research. The implementation of the peer-led ROSE model with strategies to improve recognition, organizational support and skill development for peer workers is a positive step in ensuring that peer workers are recognized as experts within their organizations, have the necessary resources and support to prevent burnout and compassion fatigue, and have opportunities for skill development [41, 51]. There is a need to make such supports available to peer workers across BC and Canada.

One limitation of this study is that it represents data from only two sites, both of which are situated in large urban centers; the competencies of peer workers who use pulse oximeters in rural areas may be different. Another limitation was that the raw data was initially coded by the academic researchers, and we recognize that some important aspects of the reality of peer workers’ lives may have been lost as a result. However, we used participatory coding in subsequent rounds and conducted data validation meetings with PRAs to mitigate this issue. Because the applied focus for this paper and the selection of Covert et al.’s framework was finalized after the interviews were conducted, the interview guide was not designed to ask about competencies. The emergent data set, therefore, did not touch on all competencies described in Covert et al (2019)’s framework in detail [21]. This, however, does not mean that peer workers who use pulse oximeters do not have those competencies; we simply may not have asked them to elaborate on those competencies. We mitigated this issue by asking for clarification on certain points related to competencies during data validation meetings. Additional research with a specific focus to create a framework of core competencies for peer workers needs to be conducted.

## Conclusion

Our study shows that peer workers who use oximeters to supplement overdose response possess several skills and competencies that are in line with those of community health workers. As such, the applicability of competency frameworks such as the one established by Covert et al. (2019) should be considered for peer workers, as the tasks they perform are vital in keeping the population they support safer from harm. The centrality of peer workers in overdose response has been consistently demonstrated and appraising their success per established competencies is essential to widely recognizing their skills. Increasing the credibility of peer workers within networks of care is integral to improve health outcomes for PWUS. Future research should focus on developing a set of core competencies for peer workers. Research to understand the perspectives of clients (PWUS) where pulse oximeters were used would also be an important next step from this work.

## Supporting information

S1 File Interview guide for the evaluation of the use of pulse oximeters in overdose response settings. (DOCX)

## Abbreviations

BC: British Columbia
COVID-19: Coronavirus Disease of 2019
Narcan: naloxone (an opioid antidote)
Nod: a back-and-forth state of being conscious and semiconscious
PRA: peer research assistant
PWUS: people who use substances
