# Supplementary material for: Core competencies of peer workers who use pulse oximeters to supplement their overdose response in British Columbia
Source: PLoS One. 2022 Sep 2;17(9):e0273744. doi: 10.1371/journal.pone.0273744 (PMC9439192; doi:10.1371/journal.pone.0273744)
Supplement: S1 File — (DOCX) [file pone.0273744.s001.docx]

**
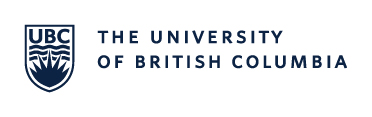

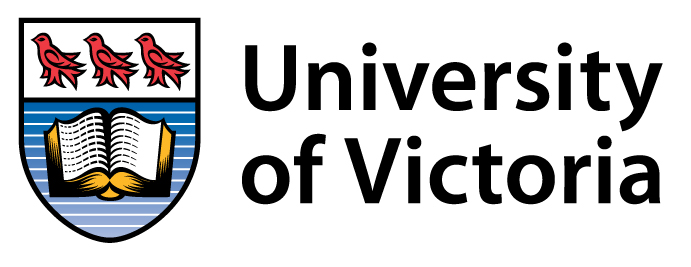
**

**PEER-2-PEER (P2P) Project: Evaluation of the Use of Oximeters in Overdose Response Settings**

**Interview Guide**

**Introduction**

Hello (interviewee),

Damian here with the Peer-2-Peer Project. I am calling about the oximeter interview.

How are you?

Are you ready to start?

The interview contains ~30-40 questions. We will start by briefly reviewing some background information on the project and then the purpose of the interview. Again, thanks so much for participating today. We value your time, input, and experience that you have to share.

**Background**

This interview is part of an evaluation being conducted by the Peer-2-Peer Project with the Harm Reduction Program at the BCCDC. The Peer-2-Peer Project is funded by Health Canada and aims to identify supports needed by people with lived and/or living experience with substance use who work in overdose response situations (i.e., Peers or experiential workers).

As you know, there have been increasing numbers of mixed overdoses involving illicit opioids and benzodiazepines or benzo-like substances. In response to this, the Peer-2-Peer Project has recently provided experiential workers at Solid Outreach and RainCity Housing with pulse oximeters to help when responding to these complex overdoses.

Pulse oximeters measure a person’s pulse rate or heart rate, and the percentage of oxygen in a person’s blood (also known as oxygen saturation). This information can help assess the effectiveness of resuscitation efforts (e.g., response to naloxone and giving rescue breaths) and determine whether more breaths are needed while waiting for paramedics to arrive. Normal oximeter readings range from 95 to 100%; values less than 90% are considered low and indicate the need for more oxygen.

The purpose of this interview is to:

- Learn about the oximeter training you received
- Understand if and how you have used the pulse oximeter
- Understand how the pulse oximeter has affected you and your overdose response skills, especially during COVID-19, and
- Get your thoughts on the pulse oximeter program

By hearing about your experience, we want to improve the pulse oximeter program and better support the vital role that experiential workers play in overdose response settings.

*(read key points of consent if necessary)*

*Are you currently being paid or is this in your time?*

*Just want to make sure we pay you appropriately.*

**TURN ON AUDIO RECORDING**

*Okay I have read the key points from the consent form. Do you consent to the interview?*

*Do you have any questions before we start the interview questions?*

**Interview Questions**

**Category 1: Quality Assurance**

**Theme A: Receipt of oximeters**

1. When did you first hear about SOLID/RainCity having oximeters?
2. When did you start using your oximeter while responding to overdoses?
3. When did you receive the oximeters?
4. How do you distribute them?
   1. Are the oximeters stored with a sign in/out sheet, etc.?
   2. Or, does each individual get their own?
   3. Are there any concerns about oximeters disappearing or going missing?

**Theme B: Training**

1. Can you please tell me about the training that you have received in overdose response in general?
   1. *Prompts*:
      1. For example, have you received training in basic life support, first aid or naloxone use?
      2. (if yes) Did this training cover material on oximeter use?
      3. Do you have any medical training or experience?
2. Can you please tell me about the training that you received on how to use an oximeter?
   1. *Prompts*:
      1. For example, who provided the training?
      2. What was the training like?
3. Do you feel that the training(s) you received was/were enough for you to feel confident to use an oximeter while responding to an overdose?
4. What additional training do you think would be helpful for you to respond to overdoses safely and effectively?

**Theme C: Process of using oximeters**

1. What, in your opinion, are the most important steps of using an oximeter during an overdose response?
2. How do you clean the oximeter?
3. I am curious about how difficult the oximeters are to use.
   1. *Prompt*
      1. Would you say they are easy, moderately difficult, or difficult to use?
      2. Why?
4. After responding to an overdose, what reporting procedures do you follow?
   1. Have you tried using the ‘Oximeter Usage Survey’ and ‘Oximeter Use Tracking Sheet’?
   2. Do you have any feedback on these forms?
      1. For example, is this too much paperwork?

**Category 2: Uptake, Utility, and Impact**

**Theme A: Uptake and utility of oximeters, and their impact on overdose responses**

1. How often do you carry an oximeter with you?
2. How many times have you used an oximeter in an overdose response?
3. How have you used an oximeter when responding to an overdose?
4. Do you think using an oximeter to monitor a person’s blood oxygen level has changed how you respond to an overdose and/or the outcomes of the overdoses you’ve responded to?
   1. If yes, how so?
   2. *Prompts*:
      1. For example, has using an oximeter helped you decide whether to provide rescue breaths?
      2. How about whether to provide naloxone or more naloxone?
      3. Do you think using an oximeter has changed the number of people that you would have given rescue breaths to? If yes, how so?
5. Do you use a pulse oximeter to measure a person’s heart rate? If so, do you think using an oximeter to monitor a person’s heart rate has changed how you respond to an overdose and/or the outcomes of the overdoses you’ve responded to?
   1. If yes, how so?
   2. *Prompts*:
      1. For example, has using an oximeter helped you decide whether to provide chest compressions?
6. Do you think using an oximeter has changed the likelihood of you calling 911 and/or the person who overdosed being sent to the hospital?
7. Have you been in any situations in which the pulse oximeter was inaccurate or didn’t work?
   1. If so, what happened?
8. We are curious about the acceptability of the oximeters from the clients’ perspective. Have any of the clients on whom you have used a pulse oximeter expressed any concerns about the pulse oximeter?
   1. If so, what did they say?
   2. What happened?

**Theme B: Impact of oximeters on experiential workers**

1. Has using an oximeter affected your **confidence** while responding to an overdose?
   1. If yes, how so?
   2. *Prompts*:
      1. For example, does being able to monitor the person’s heart rate or blood oxygen level make you feel more informed/better prepared to respond?
      2. Does it make you feel calmer or less anxious?
   3. How is your confidence affected when the person doesn’t wake up right away?
2. Has using an oximeter affected your ability to respond to an overdose?
   1. If yes, how so?
   2. *Prompts*:
      1. For example, do you feel more equipped or more prepared to respond to an overdose?
      2. Do you feel more skillful?
3. Does using an oximeter change the amount of time or energy that you need to respond to an overdose?
   1. *Prompts*:
      1. For example, how much time does it take to find, turn on, apply, and read?
      2. Has it ever delayed your use of naloxone, or delayed you calling for help?

**Theme C: Effect of oximeters on experiential workers during COVID-19**

1. Do you find it helpful to use a pulse oximeter while responding to an overdose during the COVID-19 pandemic?
   1. If yes, how so?
2. As you know, there has been controversy about giving rescue breaths during COVID-19. Has using an oximeter changed your decision-making about giving rescue breaths?
   1. If yes, how so?
   2. *Prompts*:
      1. For example, when to give rescue breaths?
      2. Or for how long?
      3. Does using an oximeter during the COVID-19 pandemic make you feel safer?
   3. If so, how has it made you feel safer?
      1. Do you have any questions, thoughts, or concerns about your risk of getting COVID-19 when you respond to an overdose?

**Category 3: Input of persons with lived experience and demographics**

**Theme A: Input of experiential workers on the oximeter program**

1. In your experience,
   1. What are the benefits of carrying an oximeter?
   2. How about the drawbacks?
2. How can experiential workers be best supported in using oximeters?
3. What are some other ways that experiential workers can be supported to respond to overdoses that involve benzodiazepines or where the person who overdosed doesn’t wake up after receiving naloxone?
4. What personal protective equipment would you like to be able to respond to overdoses safely and effectively?
5. Do you have any other suggestions to improve the oximeter program or anything else you’d like to add that you may not have had a chance to say earlier?

**Theme B: Demographics of the interviewee**

1. Finally, I would like to ask for some information about you. Having this information will help the research process.
   1. How old are you?
   2. What gender do you identify as?
   3. Do you work at Solid or RainCity?
      1. If RainCity, which location (Vancouver, Maple Ridge or Coquitlam)?
   4. What type of overdose responder are you (e.g., OPS worker, witness substance use, street patrol, outreach, etc.)?
   5. How long have you been working in overdose response?

**Conclusion**

Alright, that concludes the interview. Thanks so much again for participating today. Your input and experience have been incredibly helpful. Please do not hesitate to contact us if you have any questions in the future.

**TURN OFF AUDIO RECORDING**
